# Spray Pyrolysis Synthesis of Li_2_O–V_2_O_5_–B_2_O_3_ Glass for the Low‐Temperature Sintering of LATP Electrolytes in Solid‐State Lithium Metal Batteries

**DOI:** 10.1002/smll.202509553

**Published:** 2025-11-21

**Authors:** Min Kim, Yeon Woo Nahm, Ju Young Kim, Kang Min Lee, Yun Chan Kang

**Affiliations:** ^1^ Department of Materials Science and Engineering Korea University Anam‐Dong, Seongbuk‐Gu Seoul 136‐713 Republic of Korea

**Keywords:** glass, NASICON‐type electrolyte, sintering aid, solid‐state lithium metal battery, spray pyrolysis

## Abstract

NASICON–type Li_1.3_Al_0.3_Ti_1.7_(PO_4_)_3_ (LATP) is a promising solid–state electrolyte for lithium metal batteries (SSLMBs) due to its high ionic conductivity and stability. However, its high sintering temperature (800–900 °C) leads to lithium volatilization, the formation of secondary phases, and considerable energy consumption. Lowering the sintering temperature is also important for the practical application of LATP in all–solid–state batteries (ASSBs), particularly for facilitating integration with LATP–cathode composites. Herein, an amorphous Li_2_O–V_2_O_5_–B_2_O_3_ (LVBO) glass powder with spherical morphology is synthesized via spray pyrolysis at 900 °C and used as a sintering aid at loadings of 0.0–1.5 wt.% to enable low–temperature liquid–phase sintering of LATP at 650 °C. The addition of LVBO glass to LATP increases the relative density from 83.45% to 91.56% and enhances the ionic conductivity from 1.68 × 10^−4^ to 3.54 × 10^−4^ S cm^−1^. The LATP pellet with 1.0 wt.% LVBO (LATP–1.0) exhibits a critical current density (CCD) of 1.25 mA cm^−2^ in symmetric lithium cells. LiFePO_4_(LFP)|LATP‐1.0|Li full cells deliver excellent rate capabilities up to 1.0 C, whereas the full cell with pristine LATP fails at 0.2 C. This study establishes strategies for simultaneously reducing the sintering temperature and enhancing the electrochemical performance of LATP for SSLMBs.

## Graphical abstract

Spray‐pyrolyzed Li_2_O‐V_2_O_5_‐B_2_O_3_ glass is synthesized and utilized as a sintering aid to enable low‐temperature liquid‐phase sintering of LATP at 650 °C, resulting in a dense and ionically conductive solid electrolyte with excellent electrochemical performance and effective dendrite suppression in solid‐state lithium metal batteries.

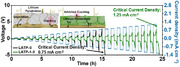

## 1 Introduction

The rapid advancement of information technology devices and electric vehicles has increased the demand for high–capacity energy storage systems such as lithium metal batteries (LMBs), which have drawn considerable attention because of their high theoretical capacity (3860 mAh g^−1^) and low reduction potential (−3.04 V vs the standard hydrogen electrode) of the lithium metal anode.^[^ ^1^, ^2^, ^3^ ^]^ However, the commercialization of LMBs is hindered by their insufficient safety, as lithium dendrites uncontrollably growing on the LMB anode can penetrate the separator to cause internal short–circuiting and thermal runaway accompanied by the decomposition of organic liquid electrolytes and release of flammable gases.^[^ ^4^, ^5^, ^6^, ^7^, ^8^ ^]^ Solid–state electrolytes (SSEs) are promising alternatives to liquid electrolytes for addressing the critical safety and performance issues of LMBs.^[^ ^9^, ^10^ ^]^ SSEs enable lithium–ion conduction through an entirely solid inorganic phase with improved thermal and mechanical stabilities by replacing flammable organic liquids and suppressing lithium dendrite growth.^[^ ^11^, ^12^, ^13^ ^]^ In addition, SSEs eliminate the need for conventional separators, enabling more compact cell designs and higher volumetric energy densities.^[^ ^14^, ^15^ ^]^

Li_1.3_Al_0.3_Ti_1.7_(PO_4_)_3_ (LATP) is a promising NASICON–type SSE because of its high ionic conductivity (10^−4^–10^−3^ S cm^−1^) and chemical and thermal stability.^[^ ^16^, ^17^, ^18^, ^19^ ^]^ This electrolyte shows low reactivity toward moisture and retains structural integrity under exposure to CO_2_ and O_2_, allowing processing under ambient conditions.^[^ ^20^, ^21^ ^]^ Furthermore, LATP exhibits electrochemical compatibility with cathode materials and a wide electrochemical stability window, supporting its application in solid–state LMBs (SSLMBs).^[^ ^22^, ^23^ ^]^ However, LATP requires sintering at high temperatures (800–900 °C) to maximize densification and ionic conductivity.^[^ ^24^ ^]^ High–temperature sintering leads to lithium loss through volatilization and the formation of secondary phases with low ionic conductivities.^[^ ^25^, ^26^ ^]^ These phases increase grain boundary resistance, suppress grain growth, and cause incomplete densification with structural defects, which degrade the microstructure and promote lithium dendrite formation.^[^ ^27^, ^28^ ^]^ In addition, the high sintering temperature poses challenges for co‐sintering LATP with cathode materials.^[^ ^29^ ^]^ Gellert et al. reported that at temperatures above 800 °C, reactions between LATP and cathode active materials lead to decomposition and interfacial phase formation.^[^ ^30^ ^]^ In the case of LFP cathodes, undesired secondary phases such as Li_2_FeTi(PO_4_)_3_ and AlPO_4_ can form, which deteriorate interfacial stability and electrochemical performance. Therefore, reducing the sintering temperature of LATP is essential for developing dense microstructures with high ionic conductivity, improving cathode–electrolyte compatibility, and enabling energy–efficient scalable fabrication of LATP‐based all–solid–state batteries (ASSBs).

The addition of a glass–type sintering aid with a low melting point has been proposed to lower the sintering temperature of LATP.^[^ ^31^, ^32^ ^]^ During the sintering process, these aids transition into the liquid phase, facilitating solid particle rearrangement, enhancing mass transport, and promoting solution precipitation to accelerate grain growth and densification at reduced sintering temperatures.^[^ ^33^, ^34^, ^35^ ^]^ Kwak et al. mixed Li_2.9_B_0.9_S_0.1_O_3.1_ glass into LATP, thus facilitating sintering and achieving an ionic conductivity of 1.50 × 10^−4^ S cm^−2^.^[^ ^32^ ^]^ The low melting point of the glass promoted grain growth during the sintering process, which in turn contributed to the observed increase in ionic conductivity. The incorporation of V_2_O_5_ into a lithium borate glass was reported to induce a structural conversion from BO_4_ to BO_3_ by breaking B–O–B linkages and forming nonbonding oxygens on the trigonal planar borate units.^[^ ^36^, ^37^ ^]^ These structural changes weakened the glass network, reduced the glass transition temperature (*T*_g_), and enhanced lithium–ion conduction by facilitating ion hopping, which has led to the application of vanadium oxide glasses as cathode materials, but using them as sintering aids has not yet been reported.^[^ ^38^, ^39^, ^40^, ^41^, ^42^ ^]^

Herein, Li_2_O–V_2_O_5_–B_2_O_3_ (LVBO) glass was prepared by using the spray pyrolysis process to produce spherical glass powder with uniform submicron–sized morphology and amorphous structure. The spherical shape and small size of these powders enabled their uniform dispersion when mixed with LATP, which minimized void formation in green pellets and facilitated heat transfer during sintering. The LATP powder was blended with LVBO glass in different weight percentages of 0, 0.5, 1.0, and 1.5 and sintered at 650 °C, a temperature lower than the typical LATP sintering range of 800–900 °C. The LVBO–modified LATP pellets exhibited high relative densities and ionic conductivities, which indicated that LVBO enhanced the sintering behavior of LATP at low temperatures. Moreover, the pellet with 1.0 wt.% LVBO glass was applied as a solid–state electrolyte to assess its practical applicability for SSLMBs.

## 2 Results and Discussion

**Scheme 1:**
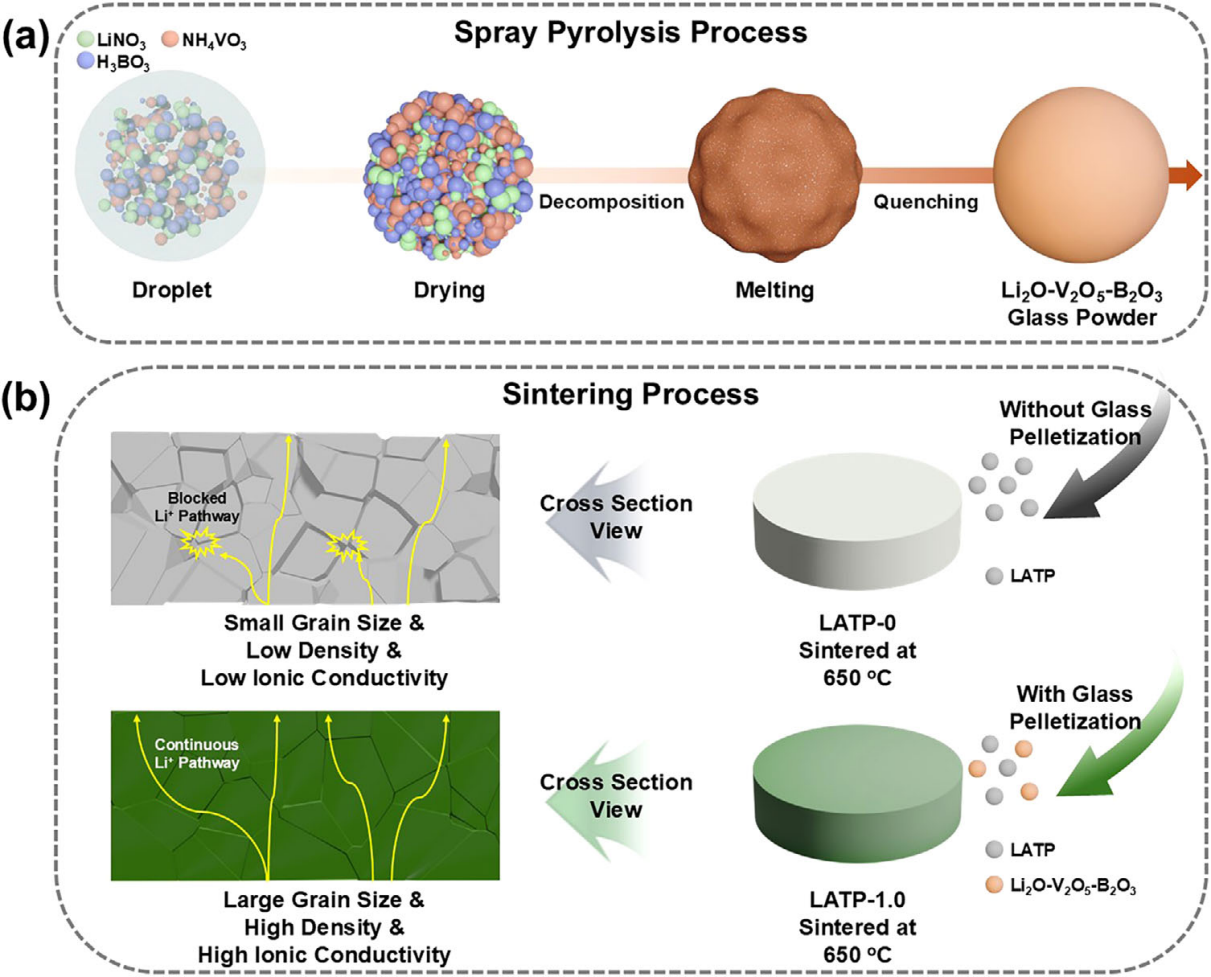
Schematic illustration of a) the synthesis process of LVBO glass via spray pyrolysis and b) a comparison of microstructural evolution after sintering without and with the addition of LVBO glass.

**Figure 1:**
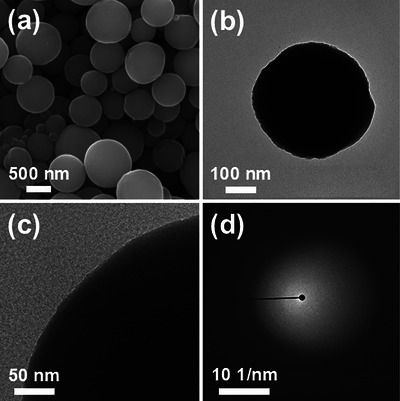
Morphologies of 900‐LVBO glass: a) SEM image, b) TEM image, c) HR–TEM image, and d) SAED pattern.

**Figure 2:**
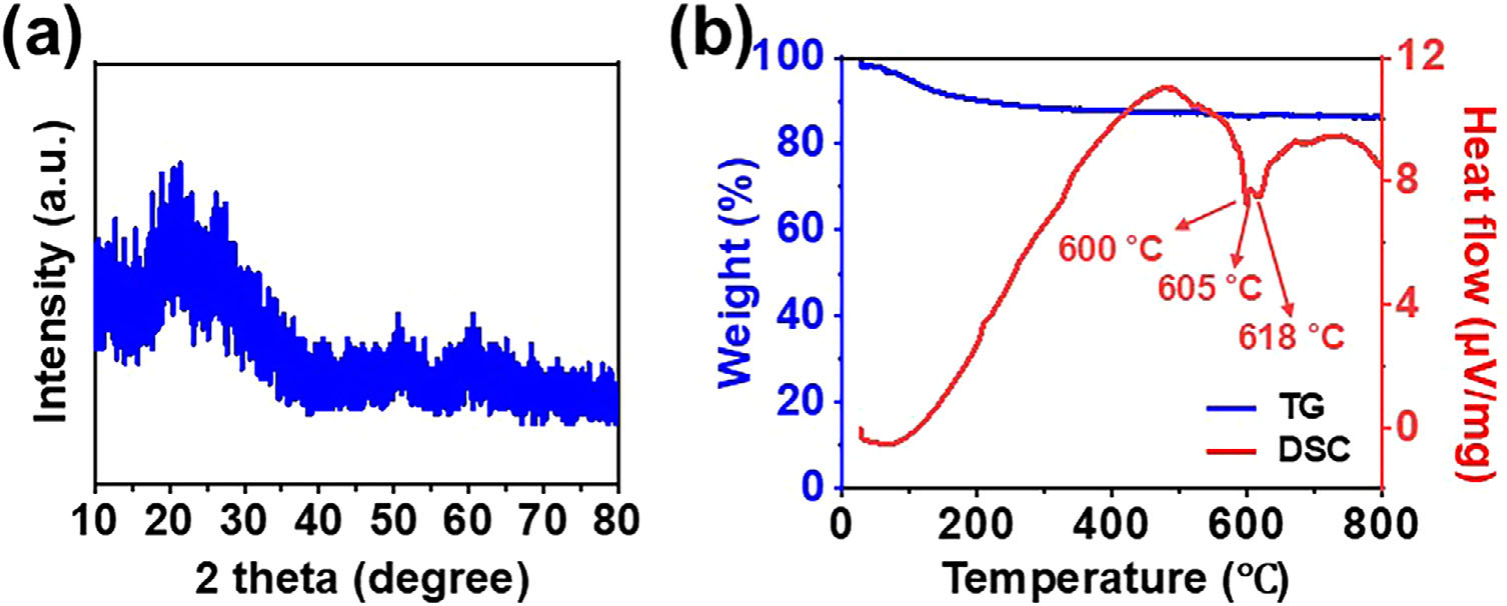
a). XRD pattern and b) TG/DSC curves of LVBO–900 glass.

The LVBO formation mechanism is illustrated in **Scheme** **1**a. During spray pyrolysis, micron–sized droplets containing lithium, vanadium, and boron salts generated using an ultrasonic nebulizer underwent drying, decomposition, and melting during their several–second residence in a hot–wall reactor and were rapidly quenched upon exiting the reactor to form submicron–sized spherical glass powder.^[^ ^43^, ^44^ ^]^ The synthesized LVBO glass was subsequently blended with commercial LATP powder and pelletized. As shown in Scheme 1b, the LATP pellet without LVBO glass exhibited a relatively porous and heterogeneous microstructure, indicative of limited sinterability at lower sintering temperatures. In contrast, the incorporation of LVBO glass is expected to facilitate liquid–phase sintering mechanisms, thereby promoting grain growth and improving densification of the LATP matrix at reduced sintering temperatures. The glass powders were prepared at 600, 700, 800, and 900 °C to investigate the LVBO formation mechanism and denoted as LVBO–600, LVBO–700, LVBO–800, and LVBO–900, respectively. The morphology and crystal structure of the LVBO microspheres were analyzed using a scanning electron microscope (SEM) and X–ray diffraction pattern (XRD), respectively. As shown in Figure S1a (Supporting Information), LVBO–600 comprised rod–like particles because of the preferential rod–shaped growth of vanadium oxide due to incomplete melting in the hot–wall reactor, as confirmed by the V_2_O_5_ peaks in the corresponding XRD pattern (Figure S2, Supporting Information).^[^ ^45^ ^]^ Enhanced melting was observed with the increasing temperature, leading to a morphological transition from rod–shaped to rough–surface spherical particles in LVBO–700 (Figure S1b, Supporting Information) and progressively smoother surfaces with minimal residual roughness in LVBO–800 (Figure S1c, Supporting Information). Broad diffraction patterns were observed above 700 °C, indicating the formation of a glass phase, as shown in Figure S2 (Supporting Information). A well–defined spherical morphology with smooth surfaces representing complete melting was attained at 900 °C (**Figure** **1**a). LVBO–900 exhibited a narrow particle size distribution, demonstrating a high degree of uniformity, with the average particle diameter equaling 0.47 µm (Figure S3, Supporting Information). As shown in Figure 1b,c, the transmission electron microscopy (TEM) and high–resolution TEM (HR‐TEM) images of LVBO–900 revealed the presence of spherical dense particles, confirming that complete melting occurred within the short duration of the spray pyrolysis process. The fuzzy rings observed in the selected area electron diffraction (SAED) pattern and the broad peak at ≈28° in the XRD pattern confirmed the amorphous nature of LVBO–900 (**Figures** 1d and **2**a). The LVBO–900 powder exhibited an ideal spherical morphology, structural homogeneity, and amorphous phase as a result of complete melting, which led to the selection of this glass as a sintering aid for further investigation. Thermogravimetric analysis (TGA)/ differential scanning calorimetry (DSC) measurements were performed by heating from room temperature to 800 °C to investigate the phase transition characteristics of LVBO–900 (Figure 2b). The TG curve revealed an initial weight loss of 9.8% up to ≈200 °C, which was attributed to the evaporation of adsorbed moisture. The DSC curve featured an endothermic peak at 600 °C corresponding to *T*_g_, with the exothermic peak at 605 °C and endothermic peak at 618 °C corresponding to the crystallization temperature (*T*_c_) and melting temperature (*T*_m_), respectively. LVBO–900 underwent a low–temperature phase transition, demonstrating its suitability as a glass additive for promoting the densification of LATP at reduced sintering temperatures. The melting and spreading behavior of LVBO–900 was further investigated by fabricating this glass powder into 8 mm–diameter disk–shaped pellets, which were then placed on alumina substrates and heated from room temperature to 700 °C at a rate of 5 °C min^−1^ (Figure S4, Supporting Information). The sample maintained its morphological stability up to 600 °C, exhibited shrinkage between 600 and 610 °C, and melted at 620 °C, in agreement with the results of DSC analysis. LVBO–900 exhibited a favorable spreading behavior at 650 °C, which facilitated LATP powder wetting and capillary force–driven rearrangement to promote grain boundary contact and densification during sintering.

**Figure 3:**
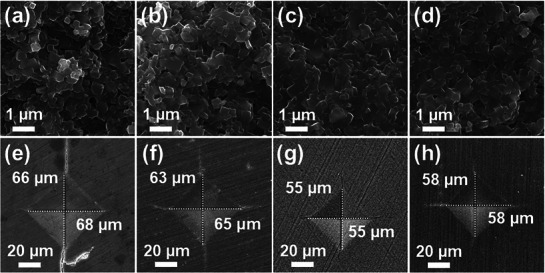
SEM images of LATP–X pellets with varying X content: a–d) Cross‐sectional microstructures of fractured surfaces and e–h) surface morphologies of Vickers indentation marks. a,e) X = 0, b,f) X = 0.5, c,g) X = 1.0, and d,h) X = 1.5.

LATP powders were mixed with varying amounts of LVBO–900 powder via ball milling to fabricate pellets. The samples were designated as LATP–X, where X represents the weight percentage of LVBO–900 (X = 0, 0.5, 1.0, 1.5). The morphology of LATP–X sintered at 650 °C was characterized by SEM analysis to evaluate grain size and densification depending on the LVBO–900 content, as shown in **Figure** **3**. In the case of LATP–0, pores were observed, implying insufficient grain growth (Figure 3a), whereas the samples with X = 0.5–1.5 showed less porous microstructures and higher relative densities (Table S1, Supporting Information). LVBO glass, which has a low melting temperature, transformed into a liquid phase during sintering, infiltrating the grain boundaries of the LATP powders. The capillary action generated by the liquid phase facilitated intimate grain interconnections and improved heat and mass transfer at the grain interfaces, thereby promoting grain growth and ceramic densification.^[^ ^33^, ^46^, ^47^ ^]^ This phenomenon enabled the sintering of LATP at a reduced temperature of 650 °C, compared to the conventional 800–900 °C. A reduction in interparticle porosity was observed for LATP–0.5, although some residual pores remained (Figure 3b). LATP–1.0 exhibited a more compact microstructure with minimally larger grains (Figure 3c). In LATP–1.5, excess LVBO resulted in a heterogeneous distribution of the glass phase, which disrupted uniform packing and increased porosity (Figure 3d). SEM–EDX elemental mapping for V, B, Al, Ti, and P was performed on the representative LATP–1.0 sample (Figure S5, Supporting Information). The mapping results revealed that all constituent elements were uniformly dispersed across the microstructure, indicating stable incorporation of LVBO into the LATP matrix. These results suggest that the LVBO–derived glass phase was homogeneously distributed throughout the structure rather than forming localized regions or compositional inhomogeneities. This uniform dispersion promoted densification by improved mass transport and grain rearrangement while maintaining the structural integrity of the LATP matrix. Vickers hardness (*H*_v_) measurements were conducted to characterize the fracture toughness, and the corresponding SEM images of the indented surfaces are shown in Figure 3e–h. A load of 9.8 N was applied to LATP–X, and *H*_v_ was calculated as:

(1) $$H_{v}=1.854P/d^{2}$$

where *P* is the load (kgf), and *d* is the mean length of the two diagonals of the cracks (mm). The Vickers hardness of LATP–0 was 4.0 GPa, whereas LVBO incorporation led to increased values of 4.4, 6.0, and 5.4 GPa for LATP–0.5, LATP–1.0, and LATP–1.5, respectively (Figure S6, Supporting Information). The *H*_v_ values followed the density order displayed in Table S1 (Supporting Information), in agreement with previous studies reporting a direct relationship between intrinsic hardness and ceramic density.^[^ ^48^, ^49^, ^50^ ^]^ This improved fracture toughness contributed to the suppression of lithium dendrite initiation and propagation in SSLMBs.

**Figure 4:**
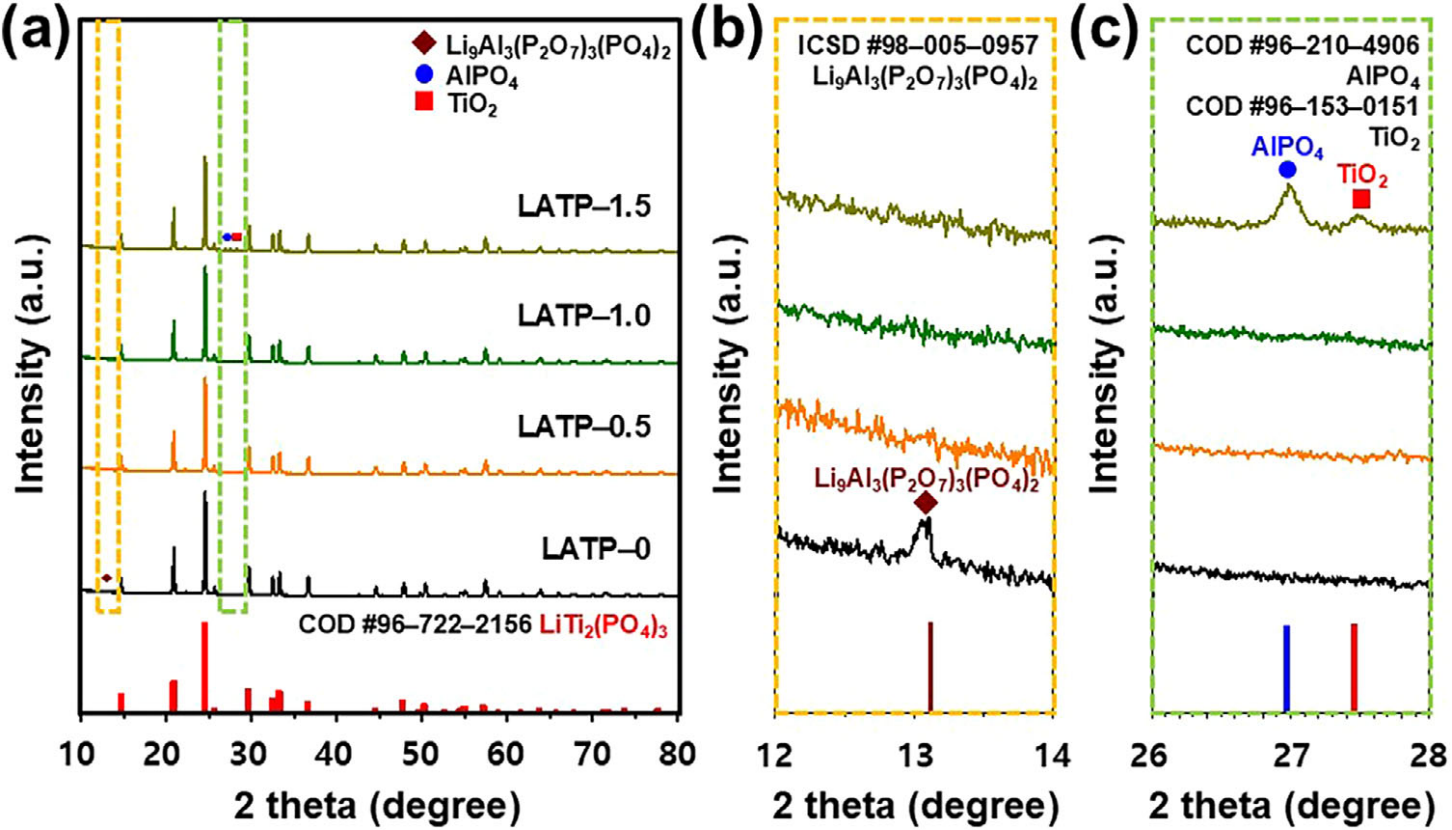
XRD patterns of LATP–X pellets with varying X content: a) in the 2θ range of 10°–80° and enlarged XRD patterns at b) 12°–14° and c) 26°–28°.

The crystal structures of LATP–X pellets sintered at 650 °C were analyzed using XRD (**Figure** **4**). The principal diffraction peaks of all samples exhibited similar half–peak widths and intensities, which demonstrated the minimal effect of LVBO on the structural composition of LATP (Figure 4a). These peaks matched the NASICON–type standard pattern of LiTi_2_(PO_4_)_3_ (LTP) COD Card No. (96–722–2156) with the R–3c space group. The enlarged XRD patterns (Figure 4b,c) revealed the presence of secondary phases. The Li_9_Al_3_(P_2_O_7_)_3_(PO_4_)_2_ phase, related to insufficient sintering, was detected in LATP–0 but not in the samples with 0.5–1.5 wt.% LVBO (Figure 4b), which suggested that LVBO enhanced the stabilization of the LTP structure during sintering and facilitated grain growth.^[^ ^51^ ^]^ In the case of LVBO–1.5, excessive liquefaction led to the decomposition of the LTP phase and formation of new secondary phases, such as TiO_2_ and AlPO_4_, increasing grain boundary resistance and hindering lithium–ion conduction (Figure 4c).^[^ ^52^, ^53^ ^]^

**Figure 5:**
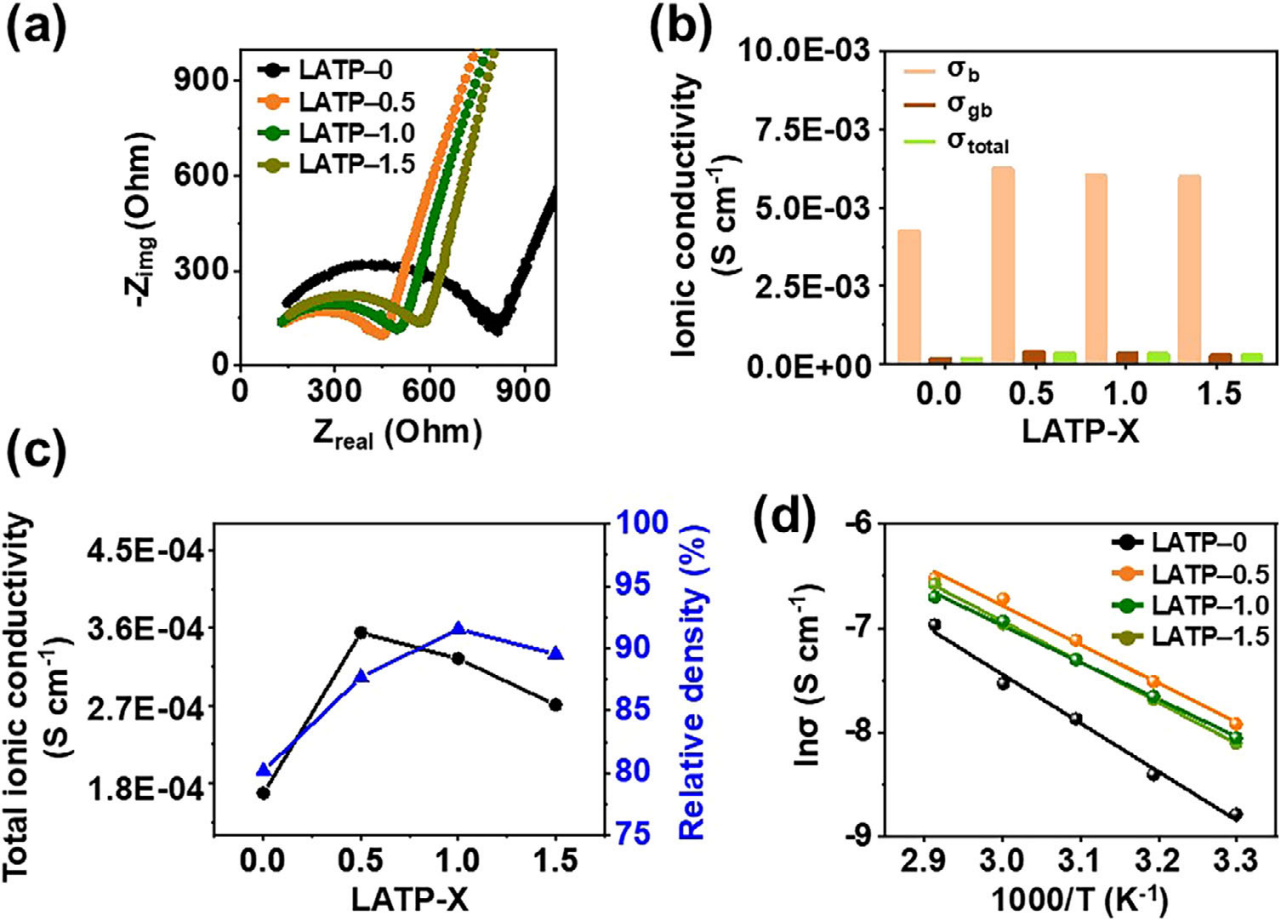
Characterization of LATP‐X pellets with varying X content: a) EIS results, b) bulk, grain boundary, and total conductivity, c) total ionic conductivity and relative density, and d) activation energy.

Electrochemical impedance spectroscopy (EIS) measurements were conducted to investigate the influence of the LVBO content on the ionic conductivity of LATP (**Figure** **5**a). The Nyquist plot of each sample exhibited a depressed semicircle at intermediate frequencies and a sloping line at low frequencies. The bulk resistance (*R_b_*) was obtained from the high–frequency intercept, and the semicircle reflected the grain‐boundary resistance (*R_gb_*). The low–frequency tail corresponds to capacitive electrode polarization described by a constant‐phase element (CPE). The less‐than‐vertical slope is attributed to interfacial inhomogeneity and surface roughness.^[^ ^54^ ^]^ The impedance components were analyzed using an equivalent circuit (Figure S7, Supporting Information). The ionic conductivity (*σ*) was determined as

(2) $$\sigma =L/\left(RS\right)$$

where *R* is the resistance obtained from the Nyquist plot, *S* is the electrolyte–electrode contact area, and *L* is the pellet thickness. The bulk conductivity (*σ_b_*), grain boundary conductivity (*σ_gb_*), and total ionic conductivity (*σ_tot_*) calculated from the EIS data are presented in Figure 5b and Table S1 (Supporting Information). These results demonstrate that ionic conduction was predominantly limited by grain boundaries rather than the bulk and that LVBO addition enhanced ionic conductivity across all compositions. The total ionic conductivities of LATP–0, LATP–0.5, LATP–1.0, and LATP–1.5 were 1.68 × 10^−4^, 3.54 × 10^−4^, 3.24 × 10^−4^, and 2.71 × 10^−4^ S cm^−1^, respectively. The highest ionic conductivity of 3.54 × 10^−4^ S cm^−1^ was achieved for LATP–0.5 and was almost twice that of LATP–0 (1.68 × 10^−4^ S cm^−1^). The correlation between relative density and ionic conductivity is shown in Figure 5c. LVBO glass incorporation substantially enhanced both properties. The ionic conductivity reached its maximum in LATP–0.5, whereas the density peaked in LATP–1.0, with both parameters minimally decreasing at higher LVBO glass contents. The discrepancy between the density and ionic conductivity trends was attributed to the dual role of LVBO during liquid–phase sintering. As observed by SEM, LVBO glass facilitated particle growth and void filling, enhancing both density and ion transport. However, excessive LVBO accumulation at grain boundaries led to the formation of nonconductive phases or disruption of continuous lithium–ion pathways within the LATP matrix, resulting in a decline in the overall ionic conductivity despite the improved relative density. Ionic conductivity was measured at different temperatures to evaluate the effect of LVBO glass addition on lithium–ion transport (Figure 5d). The activation energy (*E*_a_) was calculated from the slope of the resulting Arrhenius plot based on

(3) $$\sigma =\sigma_{0}exp(-E_{a}/\left(k_{b}T\right)$$

where *σ* is the ionic conductivity, *σ*_0_ is the pre–exponential factor, *k*_B_ is the Boltzmann constant, and *T* is the absolute temperature. The *E*_a_ values for LATP–0, LATP–0.5, LATP–1.0, and LATP–1.5 were determined as 0.43, 0.35, 0.34, and 0.37 eV, respectively, and were comparable with those previously reported for LATP (≈0.33 eV).^[^ ^55^, ^56^ ^]^ The *E*_a_ values decreased upon LVBO glass addition, implying enhanced lithium–ion transport in the glass–modified LATP ceramics. A comparison with previously reported LATP materials incorporating various sintering aids demonstrated that the LVBO system achieved superior densification, comparable ionic conductivity, and *E*_a_ at a low sintering temperature (Table S2, Supporting Information).

**Figure 6:**
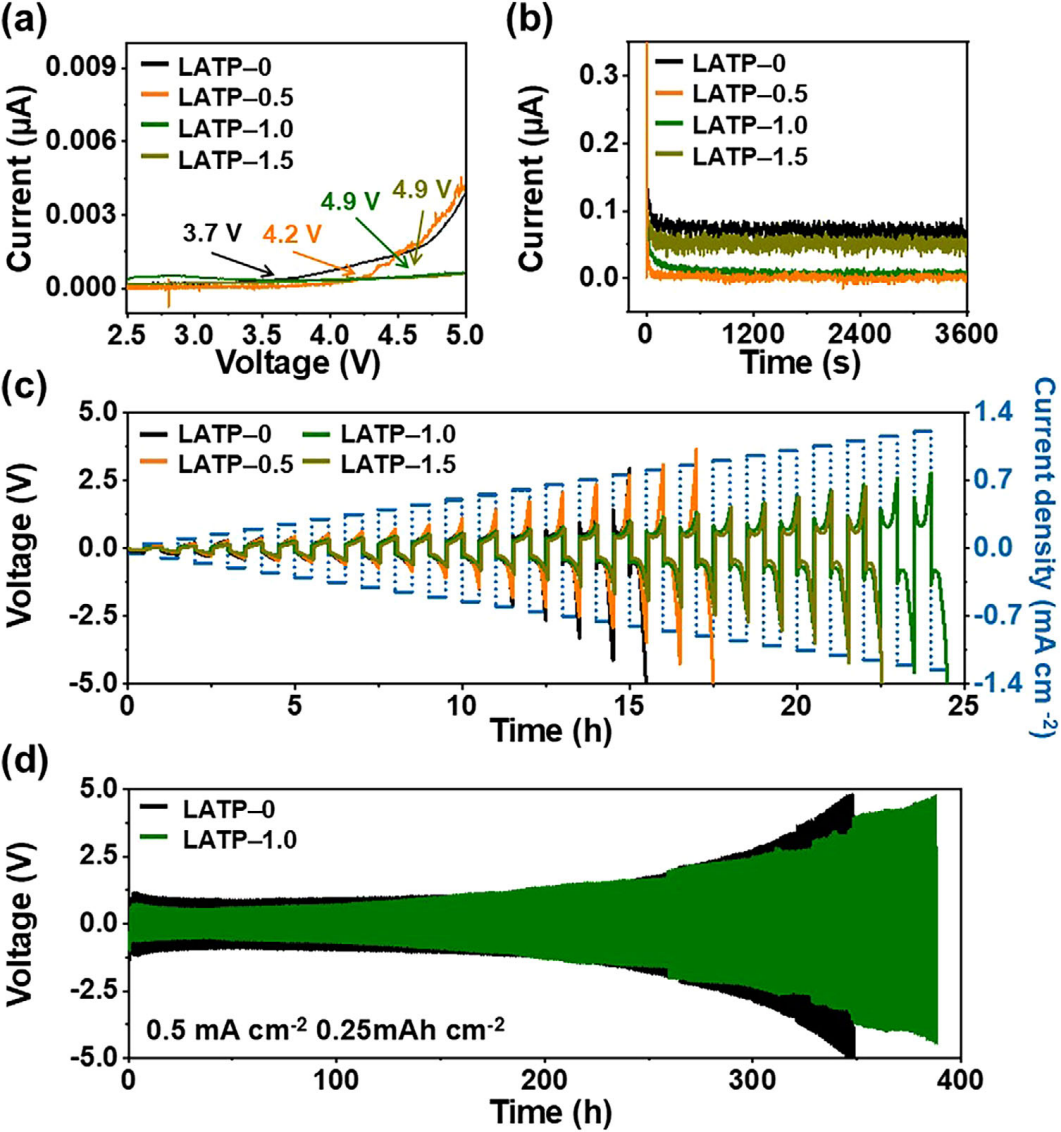
Electrochemical stability and performance of LATP–X electrolytes: a) LSV curves of Li|LATP–X|SS cells, b) DC polarization curves of Au|LATP–X|Au cells, and symmetric Li|LATP–X|Li cell performance: c) CCD tests and d) long–term cycling stability. a–c) X = 0, 0.5, 1.0, 1.5, and d) X = 0, 1.0.

**Scheme 2:**
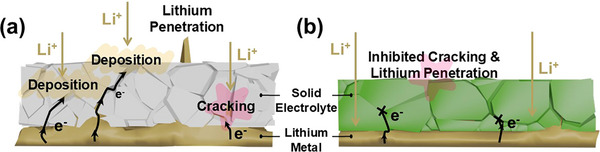
Schematic illustration of a) LATP‐0 and b) LATP‐1.0 solid electrolytes.

To further assess the applicability of LATP–X as solid electrolytes for SSLMBs, we examined their electrochemical stability by linear sweep voltammetry (LSV) in the voltage range of 2.5–5.5 V vs Li/Li^+^ at a scan rate of 0.1 mV s^−1^, as shown in **Figure** **6**a. The oxidative decomposition potential of LVBO–0 equaled 3.7 V. In contrast, the incorporation of LVBO glass enhanced anodic stability, and the oxidation potentials of LATP–0.5, LATP–1.0, and LATP–1.5 increased to 3.9, 4.9, and 4.9 V, respectively. This improvement was attributed to the suppression of interfacial reactions between LATP and lithium, which mitigated Ti^4+^/Ti^3+^ reduction and enhanced electrochemical stability. A low electronic conductivity is essential for suppressing lithium dendrite formation, as electron leakage through SSEs induces uncontrolled lithium deposition and internal short–circuiting.^[^ ^57^, ^58^ ^]^ The direct–current (DC) polarization method was used to evaluate the electronic conductivity of the LATP–X pellets (Figure 6b). A constant voltage of 0.2 V was applied to the Au|LATP–X|Au structure, where the Au electrodes were used as blocking electrodes. The current decreased with the increasing polarization time and reached a steady value (*I*), which was used to calculate the electronic conductivity (*σ_e_*) using Ohm's law

(4) $$\sigma_{e}=It/\left(AU\right)$$

where *U* is the applied voltage, *t* is the pellet thickness, and *A* is the electrode area. The *σ*_e_ of LATP–0, LATP–0.5, LATP–1.0, and LATP–1.5 equaled 4.7 × 10^−7^, 4.0 × 10^−8^, 5.5 × 10^−8^, and 3.8 × 10^−8^ S m^−1^, respectively. Although vanadium oxide–containing glasses are generally associated with increased electronic conductivities due to the presence of mixed–valence vanadium states, the LATP–LVBO composites prepared herein maintained a low electronic conductivity of 10^−8^ S m^−1^, which was four orders of magnitude lower than the total ionic conductivity.^[^ ^59^ ^]^ X‐ray photoelectron spectroscopy (XPS) revealed that both the LVBO glass and the LATP–1.0 composite exhibited a dominant V^5+^ state (Figure S8a,b, Supporting Information). The V 2p region revealed peaks assigned to V^5+^ at 517.2 eV for V 2p_3/2_ and 524.5 eV for V 2p_1/2_, while LATP–1.0 additionally exhibited weak shoulders ≈516.4 eV for 2p_3/2_ and 523.5 eV for 2p_1/2_ corresponding to V^4+^, indicating a negligible mixed‐valence fraction.^[^ ^60^ ^]^ The wider bandgap of V_2_O_5_ relative to other vanadium oxides suggests that no significant increase in electronic conductivity is expected for the V^5+^‐dominant LATP–1.0.^[^ ^61^ ^]^ This finding confirms that the incorporation of LVBO preserved the electronic insulation essential for solid electrolyte applications. The lithium dendrite formation mechanism in solid electrolytes was illustrated in **Scheme** **2** based on their microstructural and electrical properties. The LATP‐0 pellet without LVBO glass exhibited a relatively porous structure, which facilitated the formation of conductive pathways along grain boundaries and residual pores, as shown in Scheme 2a. These defects served as preferential channels for electronic leakage, enabling localized electron accumulation that reduced lithium ions to metallic lithium. In contrast, the LATP‐1.0 pellet prepared with LVBO glass exhibited a densely packed microstructure with large grains, which suppressed dendrite formation by facilitating uniform lithium‐ion transport (Scheme 2b).

The galvanostatic cycling tests were performed on Li|LATP–X|Li symmetric cells to evaluate the effect of adding LVBO glass on cell electrochemical performance and stability. The critical current density (CCD) tests were conducted to determine the maximum sustainable current density, which is a crucial factor indicating the ability of a given SSE to withstand lithium dendrite penetration (Figure 6c). The current density gradually increased from 0.05 mA cm^−2^ in increments of 0.05 mA cm^−2^ with a fixed cycling period of 0.5 h per step. The CCDs of LATP–0, LATP–0.5, LATP–1.0, and LATP–1.5 were 0.75, 0.85, 1.25, and 1.10 mA cm^−2^, respectively. LATP–1.0 exhibited the highest CCD value of 1.25 mA cm^−2^ because of its highest relative density and hardness, demonstrating an enhanced ability to suppress lithium dendrite growth at high current densities. In this case, microstructural enhancement compensated for the minimal decrease in ionic conductivity, as the increased density not only reduced the number of internal lithiated sites, such as pores and voids, but also increased the mechanical hardness of the electrolyte, improving its resistance to stress and mechanical damage caused by lithium dendrite growth.^[^ ^62^, ^63^ ^]^ Long–term cycling tests were conducted to evaluate durability at a current density of 0.5 mA cm^−2^ and capacity of 0.25 mAh cm^−2^ (Figure 6d). The Li|LATP–0|Li cell exhibited a high initial voltage polarization of 2.4 V and failed in less than 350 h due to rapidly increasing voltage polarization, whereas the Li|LATP–1.0|Li cell showed a lower initial polarization of 1.7 V and sustained cycling for over 388 h. In addition, we conducted an additional long–term symmetric test at a lower current density of 0.05 mA cm^−2^ and capacity of 0.025 mAh cm^−2^ (Figure S9, Supporting Information). Under these conditions, the LATP–1.0 cell operated for more than 1000 h with the polarization increased gradually from 0.20 to 1.64 V. To further verify the electrochemical stability of LATP–0 and LATP–1.0, Li|LATP–0|Li and Li|LATP–1.0|Li symmetric cells were cycled at 0.5 mA cm^−2^ with 0.25 mAh cm^−2^, and surface morphology, impedance, and the Ti chemical state were analyzed by ex situ SEM, EIS, and XPS after different cycle numbers. The symmetric cells were disassembled before cycling and after the 1st, 25th, and 50th cycles, and the surfaces of the solid electrolytes were examined by SEM. The obvious differences in surface morphology were observed between LATP–0 and LATP–1.0. The surface of LATP‐0 exhibited early formation of globular lithium deposits on a roughened surface, which developed into moss‐like dendrites with extended cycling (Figure S10a–d, Supporting Information). In contrast, LATP–1.0 maintained a smoother surface without developing continuous lithium dendrites, even after 50 cycles (Figure S10e‐h, Supporting Information). This morphological difference suggests that the incorporation of LVBO glass stabilized the lithium/LATP interface and suppressed dendritic lithium penetration during cycling. The impedance spectra of the Li|LATP–0|Li and Li|LATP–1.0|Li cells recorded before cycling and after the 1st, 5th, 10th, 25th, and 50th cycles correlated well with the galvanostatic cycling results, providing further insights into the interfacial stability and durability of the corresponding solid electrolytes. The Nyquist plots of Li|LATP–0|Li and Li|LATP–1.0|Li at different cycles are shown in Figure S11a,b (Supporting Information), respectively. The equivalent circuit used for fitting included *R_b_*, *R_gb_*, electrolyte/electrode interfacial resistance (*R_int_*), and Warburg impedance to account for lithium–ion diffusion (Figure S12, Supporting Information). The fitting results of total resistance (*R_tot_*), which is the sum of *R_b_*, *R_gb_*, and *R_int_*, are summarized in Figure S11c (Supporting Information). The values of *R_tot_* for LATP–0 were 3.1, 9.4, 9.7, 8.0 4.8, and 5.6 kΩ in the fresh state and at cycles 1, 5, 10, 25, and 50, respectively. The abnormal rise in *R_tot_* at the first cycle was attributed to the low relative density of LATP–0, which disrupted continuous lithium–ion pathways and induced local current constriction, accelerating interfacial Ti reduction and uneven lithium propagation.^[^ ^64^, ^65^, ^66^ ^]^ The value of *R_tot_* decreased to 4.8 kΩ at cycle 25 due to partial densification of the porous LATP network by lithium filling and a mixed–conducting interphase, but increased to 5.6 kΩ at cycle 50 as lithium dendrite growth intensified and the interfacial contact degraded. In contrast, LATP–1.0 exhibited a lower and smoother evolution of *R_tot_*, rising from 2.2 kΩ in the fresh state to 2.3, 2.6, 2.8, 3.5, and 3.9 kΩ at cycles 1, 5, 10, 25, and 50, respectively. The absence of an abnormal resistance spike after the first cycle suggests that LATP–1.0 formed a more stable initial interface with lithium metal than in LATP–0. The chemical states of Ti in pristine and cycled LATP–0 and LATP–1.0 electrolytes were analyzed by XPS using symmetric Li|LATP–X|Li (X = 0, 1.0) cells after 10 cycles, as shown in Figure S13 (Supporting Information). For both LATP–0 and LATP–1.0, peaks were observed at binding energies of 465.1 and 459.3 eV, corresponding to the Ti 2p_1/2_ and Ti 2p_3/2_ of Ti^4+^ (Figure S13a,b, Supporting Information). After cycling, two additional peaks appeared at lower binding energies of 463.6 eV (Ti 2p_1/2_) and 458.3 eV (Ti 2p_3/2_), indicating the presence of Ti^3+^ resulting from the partial reduction of Ti^4+^, as depicted in Figure S13c,d (Supporting Information).^[^ ^67^, ^68^ ^]^ Although both cycled samples displayed Ti^3+^ peaks, the relative area corresponding to Ti^3+^ in LATP–0 was significantly larger than that in LATP–1.0, suggesting that Ti^4+^ reduction was suppressed in the LATP–1.0 sample. Comparative ex situ SEM, EIS, and XPS results demonstrate that the LVBO glass additive enhanced interfacial stability and mitigated lithium dendrite propagation during repeated plating and stripping.

**Figure 7:**
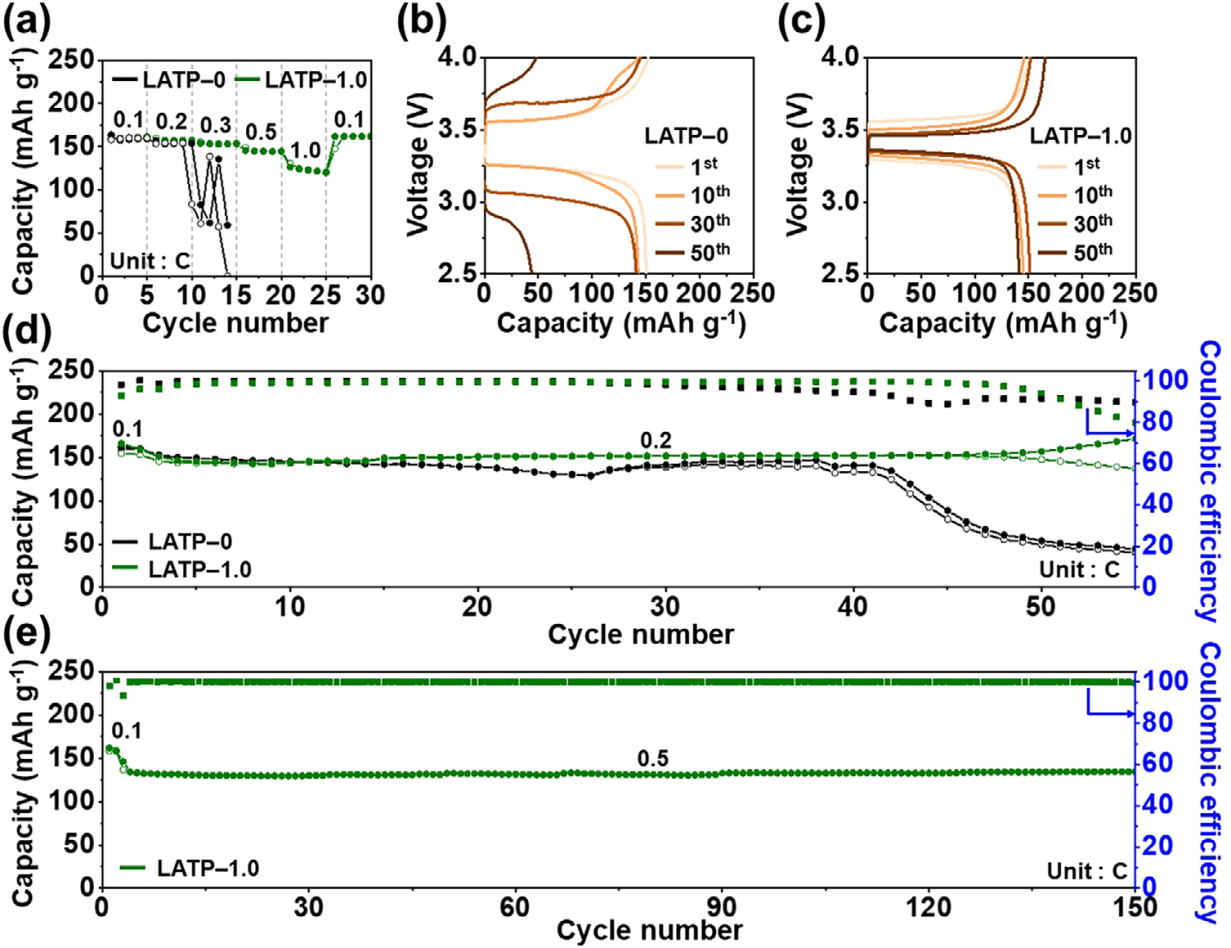
Electrochemical performance of LFP|LATP–X|Li cells. a) Rate capability of X = 0 and 1.0. Charge–discharge profiles at 0.2 C b) for X = 0 and c) X = 1.0. Long‐term cycling d) at 0.2 C for X = 0 and 1.0, and e) at 0.5 C for X = 1.0.

Full cells of LFP|LATP–0|Li and LFP|LATP–1.0|Li were assembled using LiFePO_4_ (LFP) and lithium metal as the cathode and anode materials, respectively, to verify the suitability of LATP–0 and LATP–1.0 for commercial SSLMBs. A small amount of the liquid electrolyte (10 µL) was added to the LATP–LFP interface to alleviate interfacial contact issues. The rate capability of the full cells was evaluated at C–rates of 0.1, 0.2, 0.3, 0.5, 1.0 C and returned to 0.1 C (1.0 C = 170 mAh g^−1^) as shown in **Figure** **7**a. The full cell with LATP–1.0 delivered specific discharge capacities of 161, 158, 153, 145, and 121 mAh g^−1^ at 0.1, 0.2, 0.3, 0.5, and 1.0 C, respectively, demonstrating an excellent rate capability. The capacity recovered to 161 mAh g^−1^ at the returning current density of 0.1 C, indicating structural and interfacial stability of the electrode‐electrolyte system after dynamic operating conditions. Under the same conditions, the LATP–0–based full cell failed to operate even at 0.2 C, which indicated the poor practical applicability of LATP–0 as a solid electrolyte due to its insufficient sintering properties at 650 °C. To elucidate the polarization behavior and reaction mechanisms within the full cells with LATP–0 and LATP–1.0, charge–discharge profiles were analyzed at a current density of 0.2 C. The LFP|LATP–1.0|Li and LFP|LATP–0|Li cells exhibited distinct polarization behaviors, as shown in Figure 7b,c. The LFP|LATP–1.0|Li cell presented an initial polarization of ≈300 mV that gradually decreased during cycling, indicating interfacial stabilization, whereas the LFP|LATP–0|Li cell displayed a higher initial polarization of 330 mV that progressively increased, accompanied by a rapid decline in discharge capacity due to interfacial degradation.^[^ ^69^, ^70^ ^]^ The long–term cycling performance at a current density of 0.2 C following two initial stabilization cycles at 0.1 C is presented in Figure 7d. The LFP|LATP–1.0|Li full cell delivered an initial discharge capacity of 145 mAh g^−1^ and maintained a capacity of 148 mAh g^−1^ after 50 cycles with an average coulombic efficiency of 99%. In contrast, the LFP|LATP–0|Li full cell showed capacity degradation from the 41st cycle onward. The LFP|LATP–1.0|Li cell was further cycled at 0.5 C following two initial stabilization cycles at 0.1 C, as shown in Figure 7e. The initial discharge capacity was 136 mAh g^−1^ and decreased to 134 mAh g^−1^ after 150 cycles, corresponding to a capacity retention of 99% and an average Coulombic efficiency close to 100%. These results demonstrate that LATP sintered at a reduced temperature of 650 °C in the presence of LVBO holds promise as an SSE for SSLMBs.

## 3 Conclusion

LVBO glass synthesized via spray pyrolysis was used as an effective sintering aid for a NASICON–type solid electrolyte (LATP). LVBO–900 was an amorphous glass powder comprising spherical particles with a uniform size distribution due to complete melting during spray pyrolysis. The incorporation of LVBO–900 into LATP enabled efficient liquid–phase sintering at a reduced temperature of 650 °C compared to the conventional 800–900 °C, leading to enhanced relative density, mechanical hardness, and ionic conductivity, as well as reduced overall fabrication costs. The voltage window remained stable despite the addition of LVBO, and DC polarization measurements verified that LATP–X maintained the essential electronic insulation properties of SSEs. Among the SSEs with different LVBO loadings, LATP–1.0 exhibited the best electrochemical performance owing to its dense microstructure and high mechanical strength, which played a key role in mitigating lithium dendrite growth and preventing lithium dendrite–induced structural failure. This study expands the applicability of LVBO as a sintering aid, establishing a foundation for low‐temperature processing and advanced cathode‐electrolyte co‐sintering for next‐generation ASSLMBS.

## 4 Experimental Section

### Sample Preparation

LVBO glass powder was synthesized via spray pyrolysis. The precursor solution, obtained by dissolving NH_4_VO_3_ (Samchun, 98.0%), LiNO_3_ (Junsei, 98.0%), and H_3_BO_3_ (Junsei, 99.5%) in distilled water to concentrations of 0.14, 0.03, and 0.03m, respectively, was nebulized using an ultrasonic spray generator with six droplet outlets operating at 1.7 MHz. The droplets were carried by air at a flow rate of 5.0 L min^−1^ into a quartz tube reactor maintained at 900 °C, and the resulting powders were collected using a bag filter. Detailed information, including fabrication of LVBO modified LATP pellets, characterization methods, and electrochemical measurements, is described in the supporting information.

## Conflict of Interest

The authors declare no conflict of interest.

## Supporting information



Supporting Information

## Data Availability

The data that support the findings of this study are available from the corresponding author upon reasonable request.

## References

[1] J. Peng , L.‐N. Wu , J.‐X. Lin , C.‐G. Shi , J.‐J. Fan , L.‐B. Chen , P. Dai , L. Huang , J.‐T. Li , S.‐G. Sun , J. Mater. Chem. A 2019, 7, 19565.

[2] D. J. Kang , M. J. Kim , Y. H. Jeong , G. H. Chang , J. Kim , S. Lee , H.‐T. Lim , J. Electrochem. Soc. 2024, 171, 050517.

[3] H. Yuan , X. Ding , T. Liu , J. Nai , Y. Wang , Y. Liu , C. Liu , X. Tao , Mater. Today 2022, 53, 173.

[4] C. Wang , B. B. Xu , X. Zhang , W. Sun , J. Chen , H. Pan , M. Yan , Y. Jiang , Small 2022, 18, 2107064. 10.1002/smll.202107064 35373539

[5] P. Wu , W. Zhou , X. Su , J. Li , M. Su , X. Zhou , B. W. Sheldon , W. Lu , Adv. Energy Mater. 2023, 13, 2203440.

[6] B. Kim , S. H. Yang , J. H. Seo , Y. C. Kang , Adv. Funct. Mater. 2024, 34, 2310957.

[7] A. Schreiber , M. Rosen , K. Waetzig , K. Nikolowski , N. Schiffmann , H. Wiggers , M. Küpers , D. Fattakhova‐Rohlfing , W. Kuckshinrichs , O. Guillon , Green Chem. 2023, 25, 399.

[8] S. Wen , Z. Sun , X. Wu , S. Zhou , Q. Yin , H. Chen , J. Pan , Z. Zhang , Z. Zhuang , J. Wan , Adv. Funct. Mater. 2025, 35, 2422147.

[9] C. Luo , M. Yi , Z. Cao , W. Hui , Y. Wang , ACS Appl. Electron. Mater. 2024, 6, 641.

[10] W. Yan , J. Su , Z. M. Yang , S. Lv , Z. Jin , J. L. Zuo , Small 2021, 17, 2005209. 10.1002/smll.202005209 33270359

[11] S. D. Lee , K. N. Jung , H. Kim , H. S. Shin , S. W. Song , M. S. Park , J. W. Lee , ChemSusChem 2017, 10, 2175. 28317277 10.1002/cssc.201700104

[12] S. Shin , S. Kim , D. S. Jung , K. C. Roh , J. Chun , Y. C. Kang , J. H. Kim , Adv. Eng. Mater. 2024, 26, 2301515.

[13] X. Zhan , X. Pang , F. Mao , J. Lin , M. Li , Y. Zhao , P. Xu , Z. Xu , K. Liao , Q. Zhang , Adv. Energy Mater. 2024, 14, 2402509.

[14] F. Öksüzoğlu , Ş. Ateş , O. M. Özkendir , G. Çelik , Y. R. Eker , H. Baveghar , Ceram. Int. 2024, 50, 31435. 10.3390/ma17153846 PMC11313301 39124510

[15] Q. Zhao , S. Stalin , C.‐Z. Zhao , L. A. Archer , Nat. Rev. Mater. 2020, 5, 229.

[16] K. Kwatek , W. Ślubowska , C. Ruiz , I. Sobrados , J. Sanz , J. Garbarczyk , J. Nowiński , J. Alloys Compd. 2020, 838, 155623.

[17] J.‐H. Yin , H. Zhu , S.‐J. Yu , Y.‐B. Dong , Q.‐Y. Wei , G.‐Q. Xu , Y. Xiong , Y. Qian , Adv. Eng. Mater. 2023, 25, 2300566.

[18] M. Y. Ali , H. Orthner , H. Wiggers , Nanomaterials 2024, 15, 42. 39791801 10.3390/nano15010042 PMC11723016

[19] M. Y. Ali , T. Chen , H. Orthner , H. Wiggers , Nanomaterials 2024, 14, 1278. 39120383 10.3390/nano14151278 PMC11314149

[20] H. Bai , J. Hu , X. Li , Y. Duan , F. Shao , T. Kozawa , M. Naito , J. Zhang , Ceram. Int. 2018, 44, 6558.

[21] Q. Xu , C.‐L. Tsai , D. Song , S. Basak , H. Kungl , H. Tempel , F. Hausen , S. Yu , R.‐A. Eichel , J. Power Sources 2021, 492, 229631.

[22] Y. Jin , C. Liu , X. Zong , D. Li , M. Fu , S. Tan , Y. Xiong , J. Wei , J. Power Sources 2020, 460, 228125.

[23] X. Han , S. Wang , Y. Xu , G. Zhong , Y. Zhou , B. Liu , X. Jiang , X. Wang , Y. Li , Z. Zhang , Energy Environ. Sci. 2021, 14, 5044.

[24] J. Yin , H. Zhang , Z. Zeng , G. Xu , P. Guo , H. Jiang , J. Li , Y.‐X. Wang , S. Yu , H. Zhu , J. Alloys Compd. 2024, 988, 174346.

[25] X. Xu , X. Jiao , D. Zhou , I. I. Yakovlev , P. V. Evdokimov , Y. Liu , V. S. Volkov , E. A. Goodilin , I. A. Veselova , V. I. Putlayev , J. Eur. Ceram. Soc. 2024, 44, 5774.

[26] T. Hupfer , E. Bucharsky , K. Schell , M. Hoffmann , Solid State Ionics 2017, 302, 49.

[27] X. Zhao , Y. Luo , X. Zhao , J. Alloys Compd. 2022, 927, 167019.

[28] W. Xiao , J. Wang , L. Fan , J. Zhang , X. Li , Energy Storage Mater. 2019, 19, 379.

[29] J. P. Beaupain , K. Waetzig , H. Auer , N. Zapp , K. Nikolowski , M. Partsch , M. Kusnezoff , A. Michaelis , Batteries 2023, 9, 543.

[30] M. Gellert , E. Dashjav , D. Grüner , Q. Ma , F. Tietz , Ionics 2018, 24, 1001.

[31] N. Rosero‐Navarro , T. Yamashita , A. Miura , M. Higuchi , K. Tadanaga , Solid State Ionics 2016, 285, 6.

[32] K. Kwatek , W. Ślubowska , J. Trebosc , O. Lafon , J. Nowiński , J. Alloys Compd. 2020, 820, 153072.

[33] K. Zou , Z. Cai , X. Ke , K. Wang , X. Tan , D. Luo , F. Huang , C. Wang , J. Cheng , R. Xiao , Ionics 2023, 29, 2665.

[34] N. C. Rosero‐Navarro , A. Miura , M. Higuchi , K. Tadanaga , J. Electron. Mater. 2017, 46, 497.

[35] K. Kwatek , W. Ślubowska‐Walkusz , J. Nowiński , A. Krawczyńska , I. Sobrados , V. Diez‐Gomez , J. Sanz , Ceram. Int. 2024, 50, 12450.

[36] N. Saetova , A. Raskovalov , B. Antonov , T. Denisova , N. Zhuravlev , J. Non‐Cryst. Solids 2020, 545, 120253.

[37] Y.‐I. Lee , J.‐H. Lee , S.‐H. Hong , Y. Park , Solid State Ionics 2004, 175, 687.

[38] L. S. Rao , J. Alloys Compd. 2019, 787, 1280.

[39] X. Lu , R. Sun , L. Huang , J. V. Ryan , J. D. Vienna , J. Du , J. Non‐Cryst. Solids 2019, 515, 88.

[40] X. Lu , L. Deng , S. A. Saslow , H. Liu , C. J. Benmore , B. P. Parruzot , J. T. Reiser , S. H. Kim , J. V. Ryan , J. D. Vienna , J. Phys. Chem. B 2021, 125, 12365. 34726409 10.1021/acs.jpcb.1c07134

[41] S. Afyon , F. Krumeich , C. Mensing , A. Borgschulte , R. Nesper , Sci. Rep. 2014, 4, 7113. 25408200 10.1038/srep07113 PMC5382707

[42] C. Park , S. Na , H. G. Park , K. Park , ACS Appl. Mater. Interfaces 2023, 15, 26985. 37226962 10.1021/acsami.3c04230

[43] J. Hartel , A. Banik , M. Y. Ali , B. Helm , K. Strotmann , V. Faka , O. Maus , C. Li , H. Wiggers , W. G. Zeier , Chem. Mater. 2024, 36, 10731.

[44] D. S. Jung , H. Y. Koo , S. E. Wang , S. B. Park , Y. C. Kang , Acta Mater. 2021, 206, 116569.

[45] Y. N. Ko , J. H. Kim , S. H. Choi , Y. C. Kang , J. Power Sources 2012, 211, 84.

[46] J. Yuan , B. Jiang , Y. Li , X. Guo , Y. E. Kwame , M. He , J. Mater. Sci. 2024, 59, 16629.

[47] S.‐P. Shen , G. Tang , H.‐J. Li , L. Zhang , J.‐C. Zheng , Y. Luo , J.‐P. Yue , Y. Shi , Z. Chen , Ceram. Int. 2022, 48, 36961.

[48] X. Hu , Y. Chen , Z. Hu , Y. Li , Z. Ling , J. Electrochem. Soc. 2018, 165, A1269 .

[49] Q. Zhou , C. Zhong , S. Wang , P. Jiang , L. Wang , X. Wang , C. Zhan , Energy Storage Mater. 2025, 74, 103932.

[50] E. Dashjav , M. Gellert , G. Yan , D. Grüner , N. Kaiser , S. Spannenberger , I. Kraleva , R. Bermejo , M.‐T. Gerhards , Q. Ma , J. Eur. Ceram. Soc. 2020, 40, 1975.

[51] M. Lyu , Y. Li , C. Zhang , W. Li , C. Yuan , S. Huo , W. Xue , Chem.‐Eur. J. 2025, 31, 202500820. 10.1002/chem.202500820 40152450

[52] G. Yan , S. Yu , J. F. Nonemacher , H. Tempel , H. Kungl , J. Malzbender , R. A. Eichel , M. Krüger , Ceram. Int. 2019, 45, 14697.

[53] M. Rumpel , L. Appold , J. Baber , W. Stracke , A. Flegler , G. Sextl , Mater. Adv. 2022, 3, 8157.

[54] P. Vadhva , J. Hu , M. J. Johnson , R. Stocker , M. Braglia , D. J. Brett , A. J. Rettie , ChemElectroChem 2021, 8, 1930.

[55] X. He , Y. Zhu , Y. Mo , Nat. Commun. 2017, 8, 15893. 28635958 10.1038/ncomms15893 PMC5482052

[56] K. Yang , L. Chen , J. Ma , Y. B. He , F. Kang , InfoMat 2021, 3, 1195.

[57] Y. Xiao , Y. Wang , S. H. Bo , J. C. Kim , L. J. Miara , G. Ceder , Nat. Rev. Mater. 2020, 5, 105.

[58] C. Chen , M. Jiang , T. Zhou , L. Raijmakers , E. Vezhlev , B. Wu , T. U. Schülli , D. L. Danilov , Y. Wei , R. A. Eichel , Adv. Energy Mater. 2021, 11, 2003939.

[59] B. Shao , Y. Huang , F. Han , Adv. Energy Mater. 2023, 13, 2204098.

[60] M. Prześniak‐Welenc , J. Karczewski , J. Smalc‐Koziorowska , M. Łapiński , W. Sadowski , B. Kościelska , RSC Adv. 2016, 6, 55689.

[61] N. Szymanski , Z. Liu , T. Alderson , N. Podraza , P. Sarin , S. Khare , Comput. Mater. Sci. 2018, 146, 310.

[62] J. Padchasri , S. Siriroj , P. Kidkhunthod , Radiat. Phys. Chem. 2025, 235, 112839.

[63] S. Sarkar , V. Thangadurai , ACS Energy Lett. 2022, 7, 1492.

[64] J. Zhu , J. Zhao , Y. Xiang , M. Lin , H. Wang , B. Zheng , H. He , Q. Wu , J. Y. Huang , Y. Yang , Chem. Mater. 2020, 32, 4998.

[65] J. Kang , Z. Hu , M. Niu , J. Wang , Z. Qi , Z. Zheng , Y. Liu , C. Jia , X. Ren , T. Yang , Carbon Energy 2025, e70031.

[66] J. Wang , L. Chen , H. Li , F. Wu , Energy Environ. Mater. 2023, 6, 12613.

[67] D. Y. Kibret , T. H. Mengesha , K. Z. Walle , Y.‐S. Wu , J.‐K. Chang , R. Jose , C.‐C. Yang , J. Energy Storage 2024, 94, 112523.

[68] T. Scheiber , B. Gadermaier , M. Finšgar , H. M. R. Wilkening , Adv. Funct. Mater. 2024, 34, 2404562.

[69] F. Mo , J. Ruan , S. Sun , Z. Lian , S. Yang , X. Yue , Y. Song , Y. N. Zhou , F. Fang , G. Sun , Adv. Energy Mater. 2019, 9, 1902123.

[70] Q. Xia , S. Yuan , Q. Zhang , C. Huang , J. Liu , H. Jin , Adv. Sci. 2024, 11, 2401453. 10.1002/advs.202401453 PMC11304316 38828654

