# Supplementary material for: Spray Pyrolysis Synthesis of Li2O–V2O5–B2O3 Glass for the Low‐Temperature Sintering of LATP Electrolytes in Solid‐State Lithium Metal Batteries
Source: Small. 2025 Nov 21;22(3):e09553. doi: 10.1002/smll.202509553 (PMC12802538; doi:10.1002/smll.202509553)
Supplement: Supplementary file 1 — Supporting Information [file SMLL-22-e09553-s001.pdf]

NANO · MICRO
small

Supporting Information

for *Small*, DOI 10.1002/smll.202509553

Spray Pyrolysis Synthesis of $Li_2O–V_2O_5–B_2O_3$ Glass for the Low-Temperature Sintering of LATP Electrolytes in Solid-State Lithium Metal Batteries

*Min Kim, Yeon Woo Nahm, Ju Young Kim, Kang Min Lee and Yun Chan Kang**

((Supporting Information can be included here using this template))

Copyright WILEY-VCH Verlag GmbH & Co. KGaA, 69469 Weinheim, Germany, 2013.

Supporting Information

Spray Pyrolysis Synthesis of $Li_2O–V_2O_5–B_2O_3$ Glass for the Low-Temperature Sintering of LATP Electrolytes in Solid-State Lithium Metal Batteries

*Min Kim[1], Yeon Woo Nahm[1], Ju Young Kim[1], Kang Min Lee, and Yun Chan Kang**

M. Kim, Y. W. Nahm, J. Y. Kim, K. M. Lee, and Prof. Y. C. Kang
Department of Materials Science and Engineering, Korea University, Anam-Dong, Seongbuk-Gu, Seoul 136-713, Republic of Korea

*E-mail: yckang@korea.ac.kr

[1]M. Kim, Y. W. Nahm and J. Y. Kim contributed equally to this work.

Keywords: NASICON-type electrolyte, sintering aid, solid-state lithium metal battery, spray pyrolysis, glass

Experimental part

Fabrication of LVBO modified LATP pellets

The obtained glass powders were blended with commercial LATP powder (AME Energy Co., Ltd.) via ball milling, and the mixtures were formed into disk–shaped pellets with a diameter of 10.0 mm at a pressure of 14.0 MPa and then sintered at 650 °C for 6 h. The resulting materials were denoted as LATP–X, where X = 0, 0.5, 1.0, and 1.5 is the loading of LVBO in the sintered mixture in wt.%.

Characterization methods

The morphologies of LVBO glass and LATP–X pellet were examined using scanning electron microscope (SEM, TESCAN, VEGA3) and field–emission transmission electron microscope (FE–TEM, TFEG, JEM–F200). The crystal structures of LVBO and LATP–X were analyzed by X-ray diffraction (XRD, X's pert PRO MPD) using Cu K_α radiation (λ = 0.154 nm) at the Korea Basic Science Institute (Daegu). A particle size analyzer (Malvern Panalytical, Mastersizer 3000) was used to determine average particle sizes. The phase transition characteristics of LVBO were studied using thermogravimetric analysis /differential scanning calorimetry (TGA/DSC, STA 449 C, NETZSCH) and a high–temperature wetting angle and stress measuring system (HKL–04–WASV, HKLAB). Fracture toughness was measured using a hardness tester (Mitutoyo, HM–200). The relative density for the sintered pellet was estimated using the size and weight of the pellet based on the theoretical density of LATP (2.94 g cm^{-3}). The chemical states of LVBO glass and LATP–1.0 were investigated using X-ray photoelectron spectroscopy with Al Kα radiation at Korea Institute of Science and Technology.

Electrochemical measurements

Resistance was extracted from electrochemical impedance spectroscopy (EIS) profiles recorded in the frequency range of 1.0 MHz to 1.0 Hz. Prior to measurements, the pellets were

polished with sandpaper, and both sides were sputter–coated with gold to block ions. For activation energy analysis, EIS measurements were conducted at 30–70 °C. For linear sweep voltammetry (LSV) measurements, lithium metal was attached to the pellet as a reference electrode, and measurements were performed between 2.5 and 5.5 V vs. Li/Li^{+} at a scan rate of 0.1 mV s^{-1} and room temperature. The electronic conductivity of the Au|LATP–X|Au pellets was studied using the direct–current (DC) polarization method. A constant voltage of 0.2 V was applied, and the Au coating was used as the blocking electrode. The Li|LATP–X|Li symmetric cells were subjected to galvanostatic cycling with a fixed cycling period of 0.5 h per step. Cycling stability tests were conducted at a constant current density of 0.5 mA cm^{-2}, and critical current density (CCD) tests were performed by increasing the current density from 0.05 mA cm^{-2} in increments of 0.05 mA cm^{-2}, with both surfaces of the solid electrolyte pellets Au-coated to optimize interfacial contact. Ex situ SEM, EIS, and XPS measurements were conducted before and after different cycle numbers at a current density of 0.5 mA cm^{-2} with capacity of 0.25 mAh cm^{-2} using Li|LATP–X|Li symmetric cells. For full cell fabrication, an $LiFePO_4$ (LFP)–containing cathode slurry was prepared by mixing LFP powder, a super–P conductive agent, and polyvinylidene fluoride (80:10:10, w/w/w) in *N*–methyl–2–pyrrolidone, cast on Al foil, and dried in a vacuum oven at 60 °C overnight. The electrode was punched into a circle with a diameter of 0.5 cm, with the LFP loading estimated at 3.1–3.6 mg cm^{-2}. A 1.0 M solution of $LiPF_6$ in a mixture of ethylene carbonate, dimethyl carbonate, and ethyl methyl carbonate (1:2:1, v/v/v) with 2 wt.% vinylene carbonate was used as liquid electrolyte. The fabricated full cells were cycled from 2.5 to 4.0 V at various current densities, and the theoretical capacity of LFP was assumed to equal 170 mAh g^{-1} at 1.0 C. All cells were fabricated as CR2032 coin–type cells and assembled in an Ar–filled glovebox prior to the electrochemical measurements.

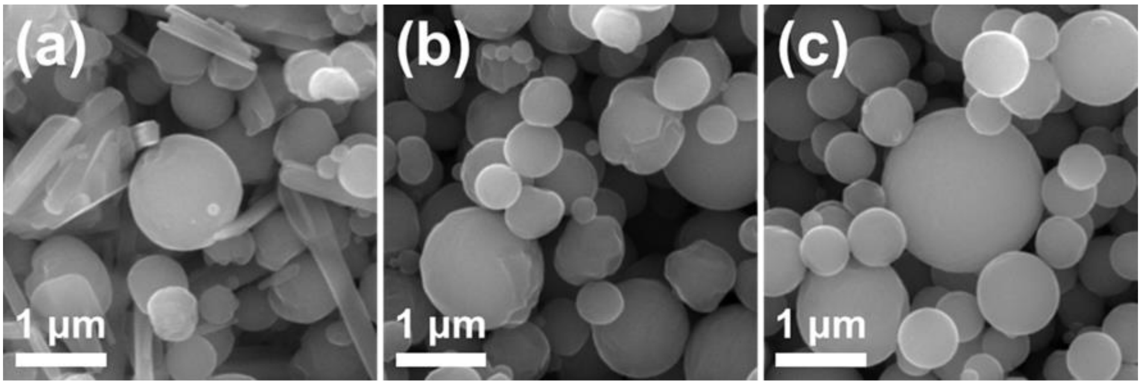

Figure S1. SEM images of LVBO glass synthesized at different temperatures: a) LVBO–600, b) LVBO–700, and c) LVBO–800.

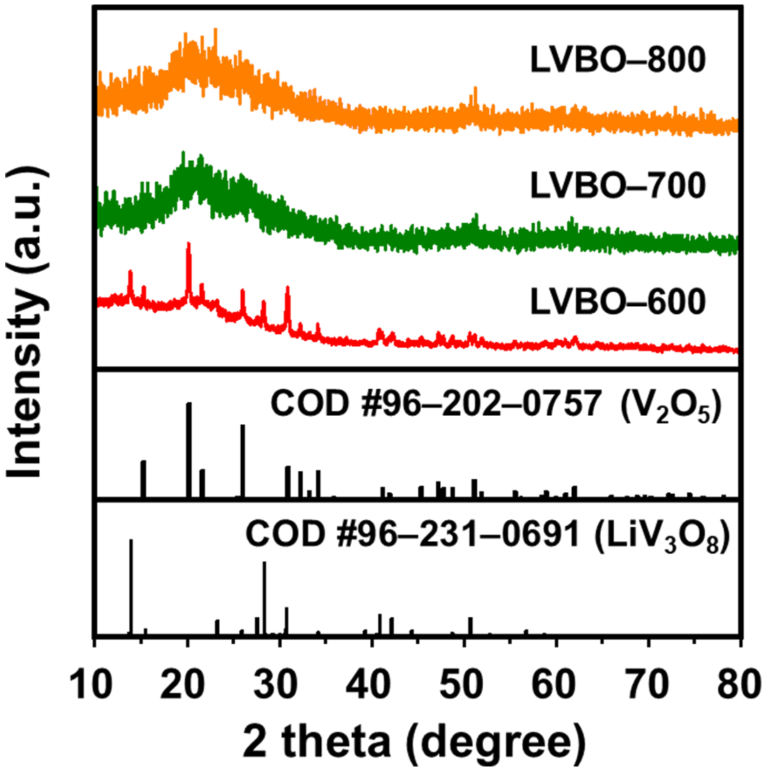

Figure S2. XRD patterns of LVBO glass synthesized at different temperatures.

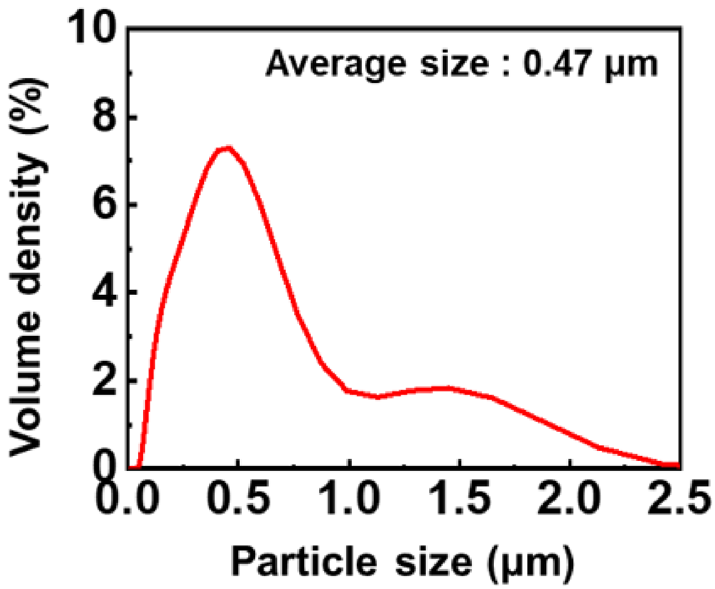

Figure S3. Particle size distribution of LVBO–900 glass.

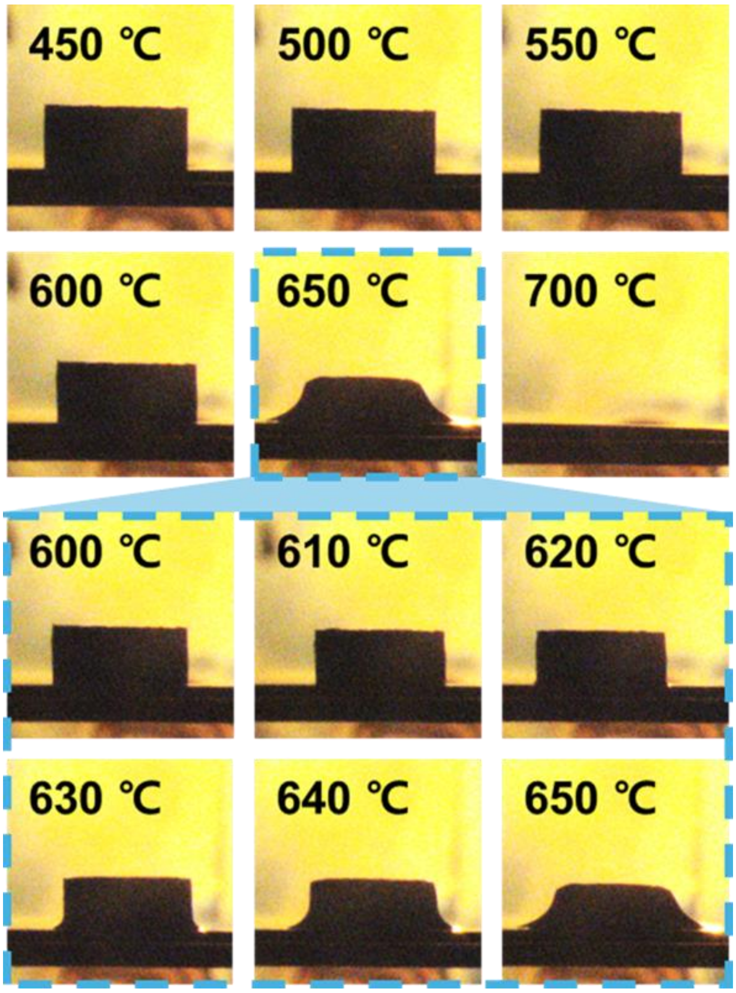

Figure S4. Digital images of LVBO–900 glass spreading behavior at different temperatures**.**

Table S1. Grain, grain boundary, and total ionic conductivity and relative density of LATP–X pellets at 30 °C

	σ_b [S cm^{-1}]	σ_{gb} [S cm^{-1}]	σ_t [S cm^{-1}]	Relative density (%)
LATP–0	4.26×10^{-3}	1.75×10^{-4}	1.68×10^{-4}	83.45
LATP–0.5	6.25×10^{-3}	3.76×10^{-4}	3.54×10^{-4}	87.67
LATP–1.0	6.05×10^{-3}	3.43×10^{-4}	3.24×10^{-4}	91.56
LATP–1.5	5.99×10^{-3}	2.84×10^{-4}	2.71×10^{-4}	89.55

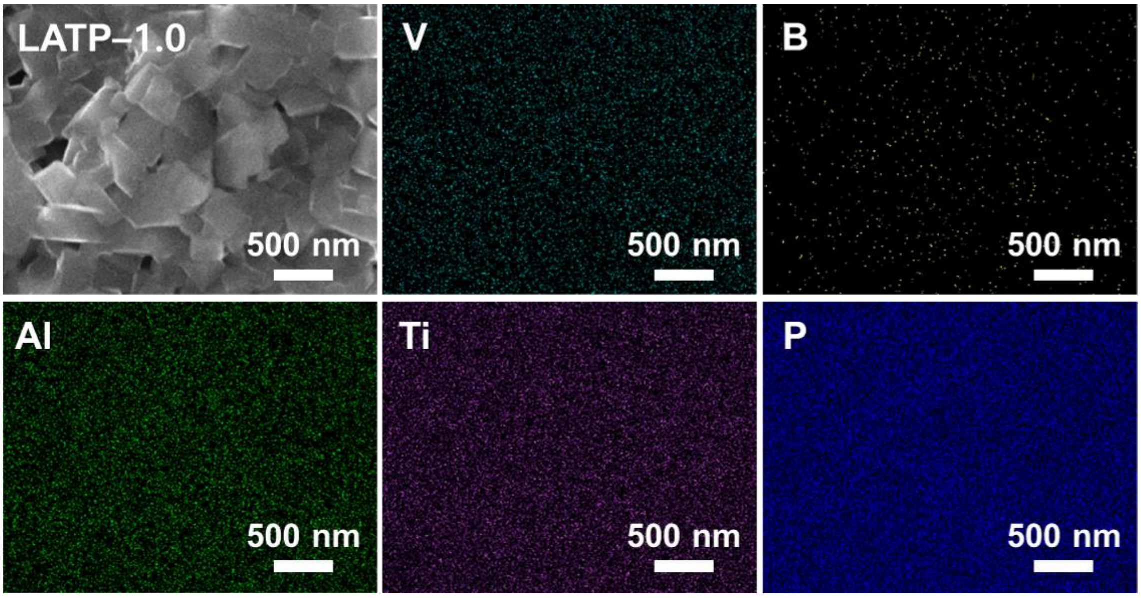

Figure S5. SEM image and corresponding EDX elemental maps (V, B, Al, Ti, and P) of LATP–1.0

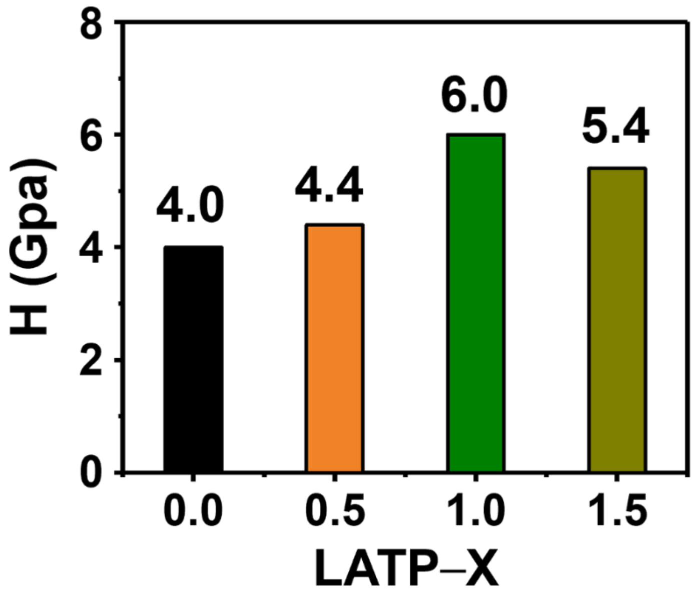

Figure S6. Vickers hardness of LATP–X pellets with varying X content.

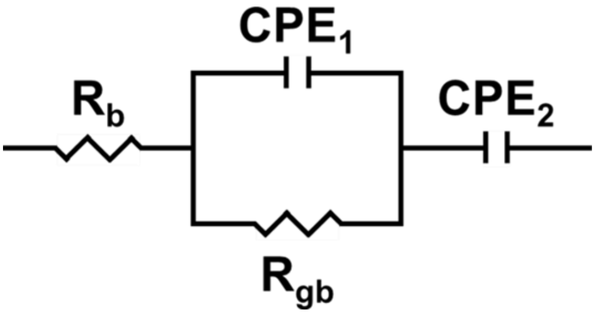

Figure S7. Equivalent circuit used for impedance analysis.

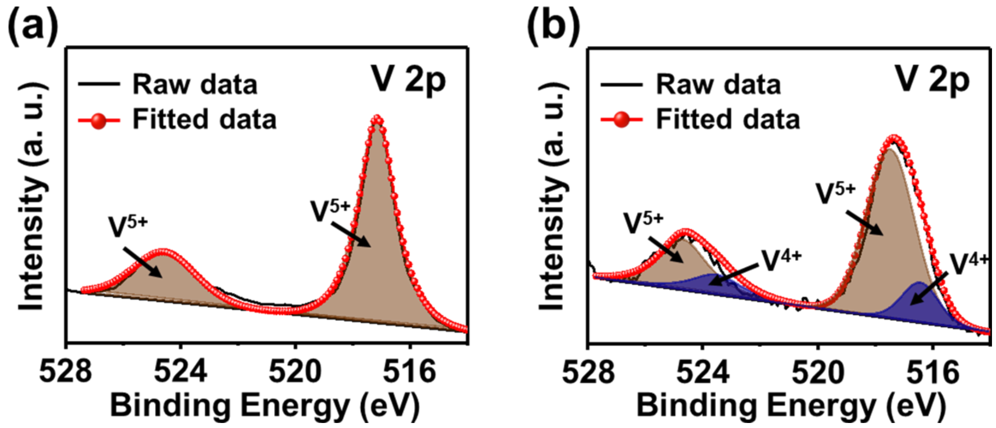

Figure S8. XPS spectra of the V 2p region for a) LVBO glass and b) LATP–1.0.

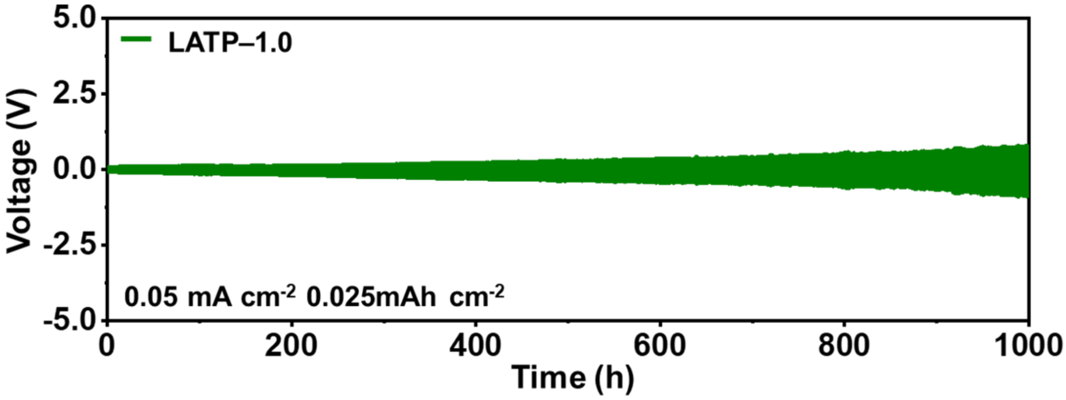

Figure S9. Long–term cycling stability of symmetric Li|LATP–1.0|Li cell at a current density of 0.05 mA cm^{-2} and capacity of 0.025 mAh cm^{-2}.

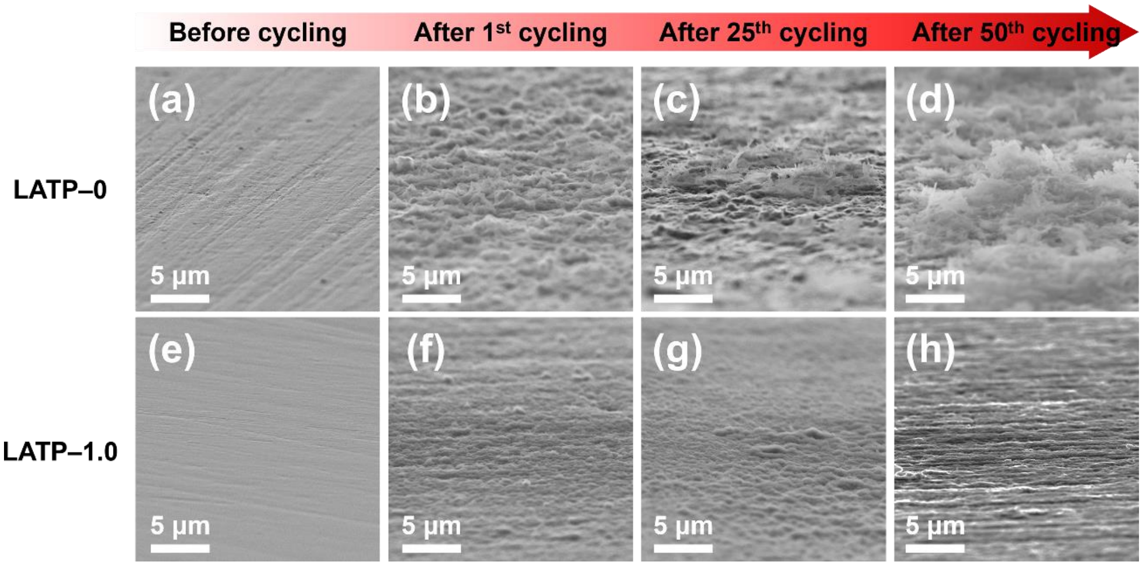

Figure S10. Ex situ SEM images of Li|LATP–X|Li symmetric cells (X = 0, 1.0) at a current density of 0.5 mA cm^{-2} with capacity of 0.5 mAh cm^{-2}: a–d) LATP–0 and e–h) LATP–1.0 after different cycling stages. a, e) fresh, b, f) 1st, c, g) 25th, and d, h) 50th cycles.

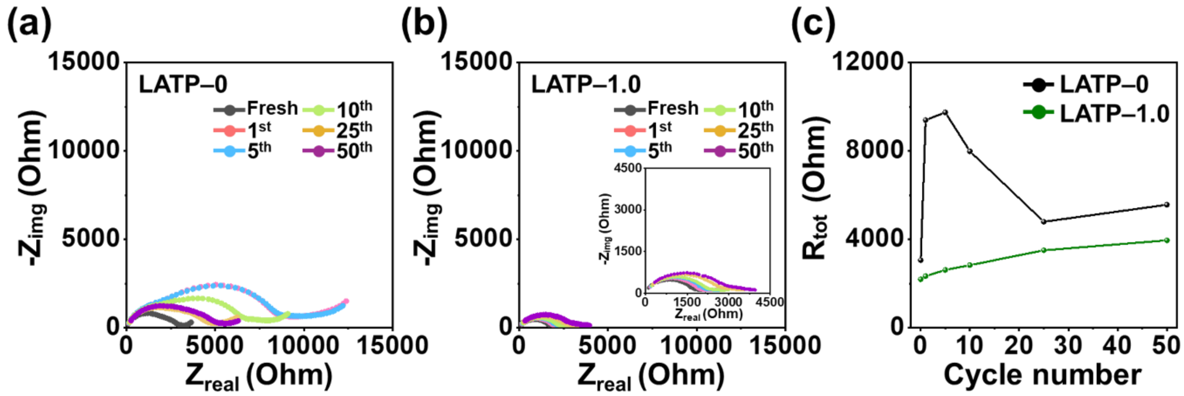

Figure S11. Li|LATP–X|Li symmetric cells (X = 0, 1.0) at a current density of 0.5 mA cm^{-2} with capacity of 0.5 mAh cm^{-2}: a) EIS spectra of LATP–0 and b) LATP–1.0 at different cycling stages. c) Total resistance versus cycle number plot for LATP–0 and LATP–1.0.

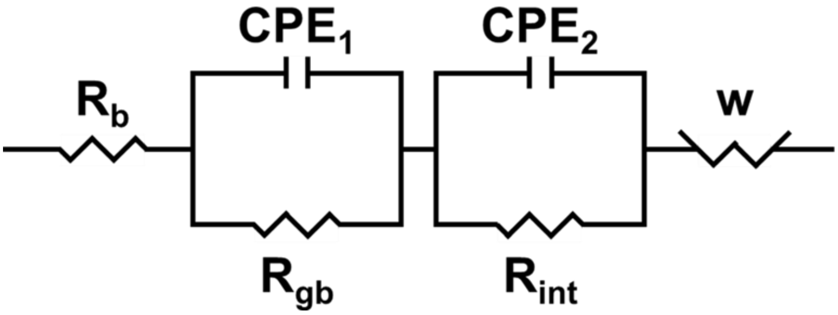

Figure S12. Equivalent circuit used for impedance analysis of symmetric cells.

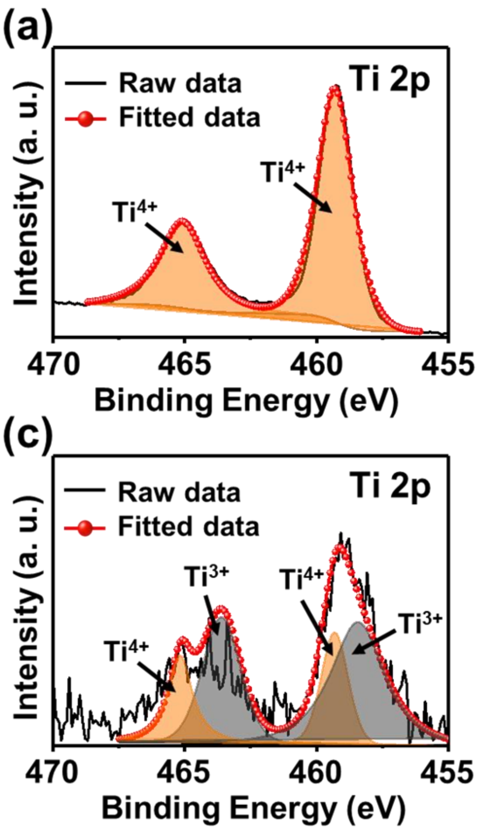

Figure S13. XPS spectra of Ti 2p for Li|LATP–X|Li symmetric cells (X = 0, 1.0) before and after cycling under a current density of 0.5 mA cm^{-2} with capacity of 0.25 mAh cm^{-2}: Pristine a) LATP–0 and b) LATP–1.0, and c) LATP–0 and d) LATP–1.0 after cycling.

Table S2. Comparison of sintering conditions, relative density, ionic conductivity and activation energy of LATP–based various glass additives of recent studies.

Samples	Sintering Conditions	Relative density (%)	Ionic Conductivity (S cm^{-1})	Activation energy (eV)	Ref
LATP–0.1 (0.75Li_2O–0.25B_2O_3)	800 °C 2 h	92.17	2.10×10^{-4}	0.32	[S1]
LATP–$LiBO_2$ (1.0 wt.%)	800 °C 2 h	97.10	3.50×10^{-4}	0.39	[S2]
LATP–TeO_2 (2.0 wt.%)	750 °C 6 h	90.64	2.00×10^{-4}	-	[S3]
LATP–0.1$Li_{2.9}B_{0.9}S_{0.1}O_{3.1}$	800 °C 2 h	94.21	1.50×10^{-4}	0.39	[S4]
LATP–B_2O_3–SiO_2–K_2O–Al_2O (2.0 wt.%)	900 °C 6 h	95.42	1.97×10^{-4}	0.36	[S5]
LATP–0.1$LiAlSiO_4$	1000 °C 2 h	93.50	2.50×10^{-4}	0.39	[S6]
LATP–SnO–P_2O_5–MgO (0.7 wt.%)	950 °C 6 h	96.72	2.45×10^{-4}	0.34	[S7]

LATP–B_2O_3 (2.0 wt.%)	850 ℃ 7 h	95.60	0.31×10^{-4}	0.26	[S8]
LATP–$LiBiO_3$ (2.0 wt.%)	880 ℃ 5 h	94.00	2.91×10^{-4}	-	[S9]
LATP–LVBO (1.0 wt.%)	650 ℃ 6 h	91.56	3.24×10^{-4}	0.34	This work

References

[S1] K. Kwatek, W. Ślubowska, C. Ruiz, I. Sobrados, J. Sanz, J. Garbarczyk, J. Nowiński, *J. Alloys Compd.* **2020**, 838, 155623.

[S2] H. Bai, J. Hu, X. Li, Y. Duan, F. Shao, T. Kozawa, M. Naito, J. Zhang, *Ceram Int* **2018**, 44, 6558.

[S3] X. Zhao, Y. Luo, X. Zhao, *J. Alloys Compd.* **2022**, 927, 167019.

[S4] K. Kwatek, W. Ślubowska, J. Trebosc, O. Lafon, J. Nowiński, *J. Alloys Compd.* **2020**, 820, 153072.

[S5] J. Kang, X. Guo, R. Gu, Y. Tang, H. Hao, Y. Lan, L. Jin, X. Wei, *J. Alloys Compd.* **2023**, 941, 168857.

[S6] K. Kwatek, W. Ślubowska-Walkusz, J. Nowiński, A. Krawczyńska, I. Sobrados, V. Diez-Gomez, J. Sanz, *Ceram Int* **2024**, 50, 12450.

[S7] J. Kang, R. Gu, X. Guo, J. Li, H. Sun, L. Zhang, R. Jing, L. Jin, X. Wei, *Ceram Int* **2022**, 48, 157.

[S8] J. Yuan, B. Jiang, Y. Li, X. Guo, Y. E. Kwame, M. He, *J. Mater. Sci.* **2024**, 59, 16629.

[S9] K. Zou, Z. Cai, X. Ke, K. Wang, X. Tan, D. Luo, F. Huang, C. Wang, J. Cheng, R. Xiao, *Ionics* **2023**, 29, 2665.
